# Effect of glycemic variability on short term prognosis in acute myocardial infarction subjects undergoing primary percutaneous coronary interventions

**DOI:** 10.1186/1758-5996-6-76

**Published:** 2014-06-30

**Authors:** Jian-wei Zhang, Ling-jie He, Shu-jun Cao, Qing Yang, Shi-wei Yang, Yu-jie Zhou

**Affiliations:** 1 Beijing Anzhen Hospital, Capital Medical University, Beijing Institute of Heart Lung and Blood Vessel Disease, The Key Laboratory of Remodeling-related Cardiovascular Disease, Ministry of Education, Beijing 100029, China; 2 Department of Emergency, Beijing Friendship Hospital, Capital Medical University, Beijing 100050, China; 3 Department of Cardiology, Beijing Daxing Hospital, Capital Medical University, Beijing 102600, China

**Keywords:** Glycemic variability-GV, ST-segment elevation myocardial infarction-STEMI, Primary percutaneous coronary interventions-p-PCI, Mean amplitude of glycemic excursions-MAGE

## Abstract

**Objective:**

Glycemic variability (GV) still remains unclear whether acute glycemic excursion has the important prognostic significance in ST-segment elevation myocardial infarction (STEMI) patients undergoing p-PCI. So our aim is to assess the prognostic value of GV in STEMI patients undergoing p-PCI.

**Methods:**

We studied 237 STEMI patients undergoing p-PCI, whose clinical and laboratory data were collected. We used a continuous glucose monitoring system (CGMS) to measure the fluctuations of blood glucose. Participants were grouped into diabetes group and non-diabetes group, and grouped into tertiles of mean amplitude of glycemic excursions (MAGE). The major adverse cardiac events (MACE) of patients was documented during in-hospital and 30-day follow-up. The relationship of MAGE and the incidence of MACE were analyzed.

**Results:**

Data from 237 subjects were incorporated into the statistical analysis, a higher MAGE level was associated with the higher peak CK-MB values (r = 0.374, P <0.01), and the higher peak cTnI values (r = 0.410, P <0.01). The rate of composite MACE by MAGE tertiles (<2.37 mmol/l, 2.37-3.65 mmol/l and >3.65 mmol/l) was 7.5% vs. 14.1% vs. 22.8%, respectively (P = 0.025); STEMI patients with a higher MAGE level had a significantly higher non-IRA revascularization compared with those with lower MAGE levels (32% vs. 15% vs. 21%, P = 0.037). Moreover, diabetic patients with higher MAGE level had significantly higher incidence of composite MACE and non-IRA revascularization, non-diabetic subjects did not show the similar results. In multivariable logistic analysis, the independent predictors of MACE were: MBG, MAGE and LVEF in diabetic subjects and were MBG and MAGE in nondiabetic subjects. Other factors were not significantly associated with MACE.

**Conclusions:**

Greater GV is associated with composite MACE and non-IRA revascularization during in-hospital and 30-day follow-up in unadjusted analyses, especially for diabetic subjects. After multivariable logistic analysis, GV remains an independent prognostic factor for composite MACE in STEMI patients undergoing p-PCI.

## Introduction

Hyperglycemia is common during acute myocardial infarction (AMI). It is also a strong predictor of mortality in AMI patients with and without a history of diabetes mellitus [1,2]. Meanwhile, some studies have revealed glycemic variability (GV), which includes both upward and downward acute glucose changes, is another important component of dysglycemia [3]. Physiological studies have suggested several mechanisms through which GV may adversely impact prognosis in the setting of AMI, including oxidative stress [4], cytokine release [5], and endothelial dysfunction [6]. In addition, GV has been associated with adverse events in other critically ill patient populations [7-10]. However, the prognostic value of GV has not been defined in patients with ST-segment elevation myocardial infarction (STEMI) undergoing primary percutaneous coronary interventions (p-PCI). Continuous glucose monitoring systems (CGMS) could provide detailed time series of consecutive observations on the underlying process of glucose fluctuations. Such detailed glucose information to patients has been shown to have positive influence on glucose control, including reduction in glucose variability, time spent in nocturnal hypoglycemia, time spent in hyperglycemia, and levels of glycosylated hemoglobin [11-13]. The assessment of GV through CGMS is now much less cumbersome. GV still remains unclear whether acute glycemic excursion has the important prognostic significance in AMI patients.

Therefore, the aim of this study was to investigate the effect of GV on short term prognosis in STEMI patients undergoing p-PCI.

## Materials and methods

### Study design and patient population

We enrolled STEMI subjects undergoing p-PCI from two major hospitals in Beijing, China (Beijing Anzhen Hospital, Beijing Daxing Hospital, Capital Medical University). This study was conducted from January 2012 to November 2013. After admission information on previous clinical history, cardiovascular risk factors, medication were collected in hospital. Moreover, laboratory and echocardiography were recorded. The inclusion criteria were: 1) confirmed admission diagnosis of STEMI undergoing p-PCI. 2) admission glucose <22.2 mmol/l, and without diabetic ketosis or nonketotic hyperosmolar coma and 3) written informed consent. The exclusion criteria included the following: 1) a history of hepatic or renal impairment, or of other diseases that can influence glucose metabolism, including malnutrition, and cancers. 2) without informed consent. Participants were grouped into diabetes group and non-diabetes group, and divided into tertiles on the basis of the mean amplitude of glycemic excursions (MAGE) level (<2.37 mmol/l, 2.37-3.65 mmol/l and >3.65 mmol/l). The study protocol was approved by the Medical Ethics Committee of Beijing Anzhen Hospital, Beijing Daxing Hospital, Capital Medical University.

### Definition of ST-segment elevation myocardial infarction (STEMI)

ST-segment elevation myocardial infarction was defined as complaints of chest pain with ECG signs compatible with acute myocardial infarction (ST-segment elevation >2 mm in precordial leads and > 1 mm in limb leads) [14]. All patients were directly transported to the catheterization laboratory on arrival, and acute coronary angiography was performed with subsequent PCI when indicated as part of the routine treatment for all STEMI patients in these institutions. The interventional strategy was at the operator’s discretion. Multivessel disease was defined as 70% or greater stenoses in at least one major epicardial vessel and 50% or greater stenoses in at least one other major vessel. All patients were pretreated with aspirin, heparin, and clopidogrel during transportation to the hospital, or these drugs were administered at the emergency ward [15].

### CGMS

All patients were equipped with CGMS (Medtronic Mini-Med, USA), and were monitored for 72 consecutive hours after p-PCI. CGMS sensor was inserted in the abdominal subcutis and calibrated every 6 hours according to the manufacturer's indications. During the study period, all subjects were provided standard mixed-meals by dietary division. The total calorie intake was 126 kJ/kg per day, with 50% carbohydrates, 15% proteins, and 35% fats. The calorie distribution between breakfast, lunch, and dinner was 20%, 40%, and 40%, respectively. Three daily meals were required to consume at time of 6:30 to 7:30, 11:30 to 12:30, and 18:00 to 19:00, respectively, and each meal had to be consumed within 30 minutes.

Patients checked their blood glucose level with a self-monitoring of blood glucose (SMBG) device (Medisafe Mini, Terumo, Japan) at least 4 times per day. The sensor measures interstitial glucose every 10 s and records the mean values at 5-min intervals. The sensor was left in place for 3 days for collection of data. Adopted from previous established criteria for optimal accuracy of the CGMS [16,17], the following criteria for optimal accuracy included: a correlation between the sensor and meter readings of at least 0.79, and a mean absolute difference of no more than 28% (when the daily range (Min-Max) of meter values ≥5.6 mmol/l); a mean absolute difference of no more than 18% (when the daily range (Min-Max) of meter values <5.6 mmol/l).

CGMS parameters: The 24-hour mean BG (24 h MBG) was calculated as mean BG level from 288 readings measured by a CGMS over 24 hours. Since measurable range of glucose by CGMS was mechanically limited from 2.2 to 22.2 mmol/L, the case showing the data out of this range was excluded from the study. The glycemic level was calculated as the mean level of 24-hour BG value (MBG), and intra-day glycemic excursions were calculated as standard deviation of BG (SDBG) and mean amplitude of glycemic excursion (MAGE). The MAGE was calculated by measuring the arithmetic mean of the differences between consecutive peaks and nadirs, provided that the differences are greater than one standard deviation (SD) of the mean glucose value [3]. Patients would maintain anti-hyperglycemic therapy as usual and be avoided glucose infusion during CGMS monitoring period. Otherwise, the patient would be excluded from the study. After monitoring for 72 hours, the recorded data were downloaded into a personal computer for analysis of the glucose profile and glycemic excursion parameters with the CGMS Software 3.0 (Medtronic MiniMed, Inc.).

### Follow-up and definitions of major adverse cardiac events (MACE)

The primary end point of this study was a composite MACE defined as the occurrence of one of the following events: cardiac death, reinfarction, repeat target vessel revascularization (TVR) after initial revascularization, or recurrent angina. Patients with more than one event were assigned the highest ranked event according to the previous list. Reinfarction was defined as recurrent chest pain with new ST segment elevation and recurrent elevation of cardiac enzymes. TVR was defined as PCI or bypass surgery performed because of reocclusion of the target vessel. Recurrent angina was defined as ischemic chest pain with either new ST segment or T wave changes at rest or on exercise testing. Malignant arrhythmia was defined as life-threatening cardiac arrhythmias with a need for resuscitation or pacemaker implantation.

The secondary end point was the incidence of non-infarct related arteries revascularization during in-hospital and 30-day follow-up.

All MACE data were adjudicated by an experienced cardiovascular physician blinded to clinical details and outcomes.

### Statistical analysis

CGM parameters were analyzed using Medtronic MiniMed CGMS Software 3.0. Data are presented as frequencies and percentages for categorical variables and mean ± SD for continuous variables. We used the *χ*^2^ to compare the categorical variables and the 2-sample *t* test for continuous variables. Intergroup differences were tested with one-way analysis of variance (ANOVA). Correlation between continuous variables was determined by Spearman correlation coefficients. MAGE was included as a continuous and as a categorized (<2.37 mmol/l, 2.37-3.65 mmol/l and >3.65 mmol/l) variable. Multivariable logistic regression analysis was used to assess whether the association between MAGE and MACE was independent of other factors. A P value of < 0.05 (two-sided) was considered significant. Data were analyzed with SPSS 21.0 (Chicago, Illinois).

## Results

Based on the inclusion criteria and the exclusion criteria, we enrolled 247 STEMI patients undergoing p-PCI, 10 cases were excluded for final analysis due to the CGMS signal interruption or not meeting the accuracy requirements. Data from the remaining 237 subjects (165 men and 72 women) were incorporated into the statistical analysis. Table 1 provided the basic characteristics of study patients. Compared to non-DM subjects, DM subjects had higher levels of Triglycerides, HbA1c, Glycated albumin(GA) (P < 0.05). MBG, MAGE and SD were higher in the DM group, compared to non-DM (P < 0.001). Patients with DM had higher incidences of multivessel disease (71% vs. 57%, P = 0.034).

**Table 1:** Clinical characteristics at baseline of study participants

	**All**	**DM**	**Non-DM**	**P-value**
Subject number	237	73	164	NA
Age (years)	54 ± 16	55 ± 17	52 ± 15	**0.174**
Males	165 (70)	50 (68)	115 (70)	0.801
BMI, kg/m^2^	25.2 ± 4.6	25.3 ± 4.5	24.8 ± 4.2	0.409
LVEF, %	53.9 ± 7.3	53.5 ± 7.1	54.2 ± 7.4	0.497
Systolic BP (mm Hg)	120 ± 21	123 ± 25	118 ± 23	0.134
Diastolic BP (mm Hg)	70 ± 9	69 ± 10	71 ± 12	0.215
Serum creatinine, mg/dL	0.80 ± 0.22	0.82 ± 0.21	0.78 ± 0.19	0.149
Total Cholesterol (mmol/l)	4.64 ± 1.05	4.56 ± 1.01	4.69 ± 1.09	0.387
Triglycerides (mmol/l)	2.23 ± 1.28	2.36 ± 1.65	1.97 ± 1.03	0.036
LDL-C (mmol/l)	2.87 ± 1.10	2.82 ± 1.13	2.91 ± 1.18	0.584
HDL-C (mmol/l)	0.98 ± 0.31	0.95 ± 0.22	1.01 ± 0.35	0.178
HbA1c (%)	6.5 ± 1.3	7.1 ± 1.2	6.1 ± 1.0	<0.001
Glycated albumin (%)	17.8 ± 7.6	21.2 ± 6.7	12.9 ± 4.5	<0.001
Peak cTNI (ng/ml)	37.3 ± 10.7	40.5 ± 11.4	36.2 ± 12.5	0.013
Peak CK-MB (ng/ml)	37.8 ± 15.6	39.4 ± 17.5	36.2 ± 16.4	0.176
FBG (mmol/l)	7.3 ± 2.8	8.8 ± 2.4	6.1 ± 1.7	<0.001
Management of DM (%)				
OHA		25 (34.2)		
Insulin only		31 (42.5)		
Insulin + OHA (s)		17 (23.3)		
**Risk factors (n, %)**				
Hyperlipidemia	93 (39)	32 (43.8)	61 (37.2)	0.334
Hypertension	95 (40)	30 (41.1)	65 (39.6)	0.832
Current smoking	106 (45)	30 (41.1)	76 (46.3)	0.453
Family history	29 (12)	8 (11)	21 (13)	0.689
Obesity	55 (23)	20 (27)	35 (21)	0.308
**CGMS parameters on the first day**				
MBG (mmol/l)	8.1 ± 2.6	10.6 ± 2.3	6.7 ± 1.1	<0.001
MAGE (mmol/l)	3.6 ± 2.0	3.9 ± 1.1	2.8 ± 1.6	<0.001
SDBG (mmol/l)	1.7 ± 1.0	2.5 ± 1.3	1.3 ± 0.7	<0.001
**Angiographic data (n, %)**				
Single vessel	92 (39)	21 (29)	71 (43.3)	0.034
Double vessels	77 (32)	27 (37)	50 (30.5)	0.324
Triple vessels	68 (29)	25 (34)	43 (26.2)	0.207
Main stem involved	32 (14)	12 (16)	20 (12)	0.378
Multivessel	145 (61)	52 (71)	93 (57)	0.034

Data given as mean ± SD or n (%).

BMI, body mass index; LVEF, left ventricular ejection fraction; LDL-C, Low density lipoprotein-cholesterol; HDL-C, High density lipoprotein-cholesterol; HbA1c, hemoglobin A1c; CK-MB, creatine kinase-MB; cTnI, cardiac troponin I; FBG, fasting blood glucose; OHA, oral hypoglycaemic agent; MBG, Mean blood glucose; MAGE, the mean amplitude of glycemic excursions; SDBG, the standard deviation of blood glucose values.

**Figure 1:**
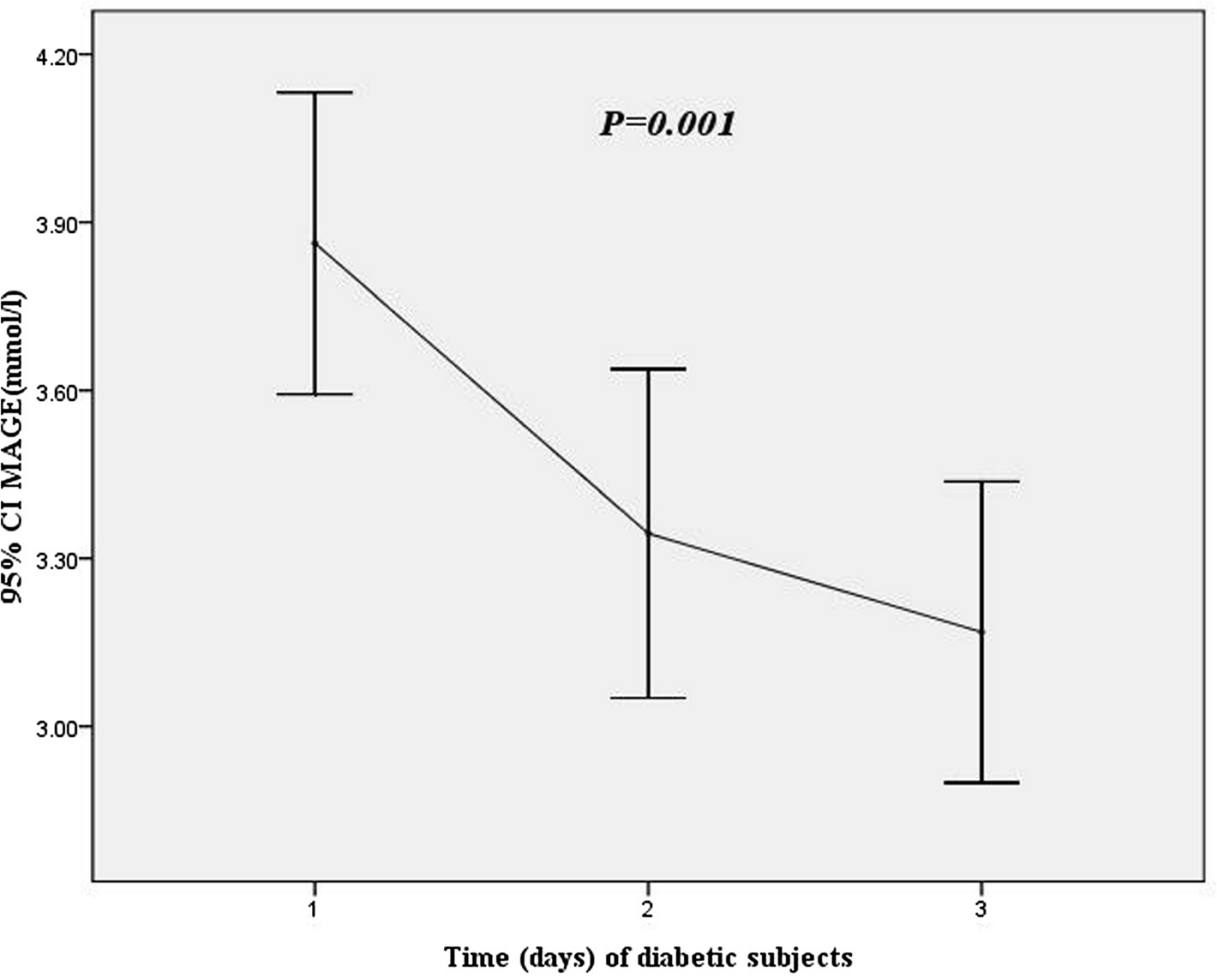
MAGE values for 3 days through using CGMS in diabetic subjects.

**Figure 2:**
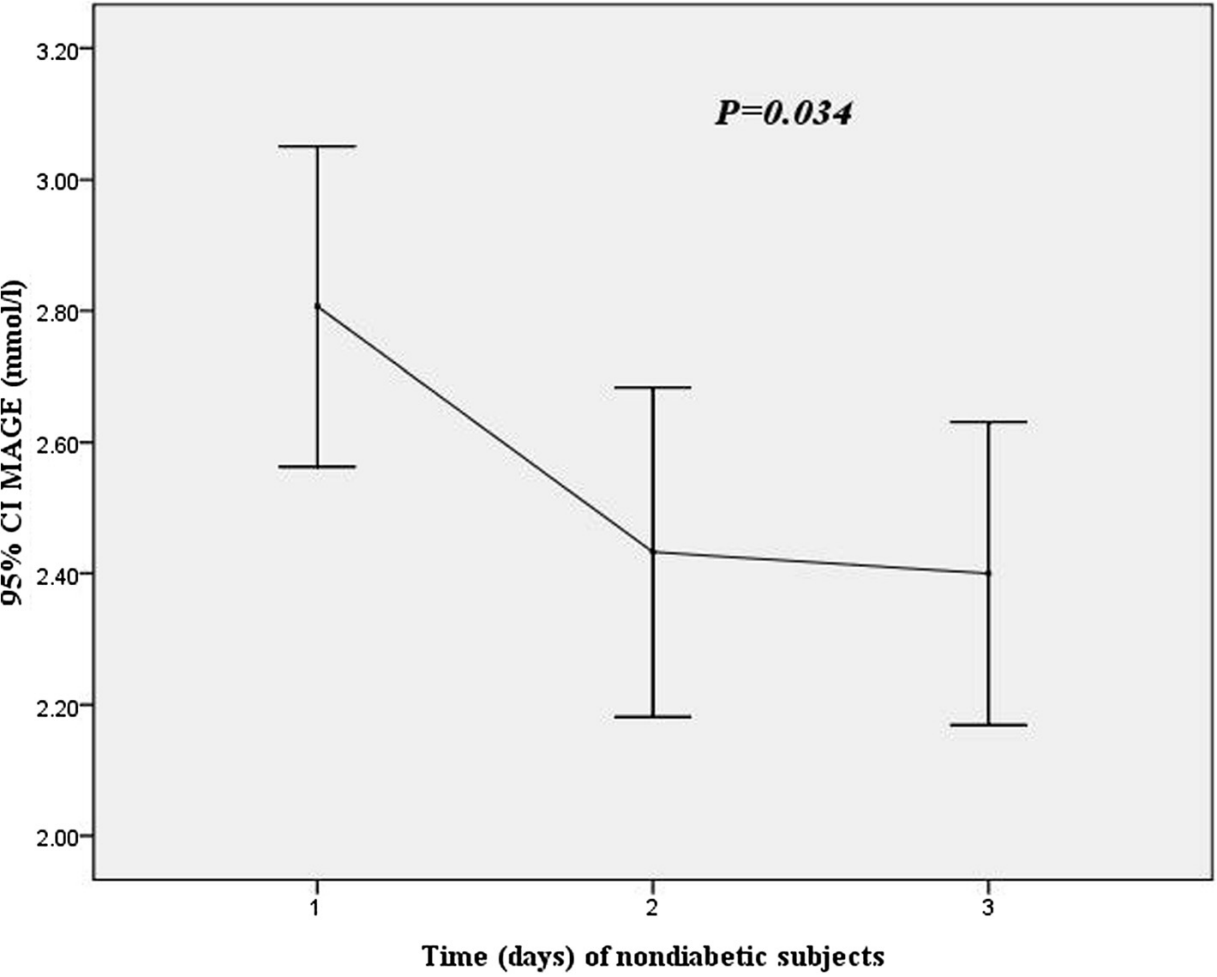
MAGE values for 3 days through using CGMS in nondiabetic subjects.

Baseline characteristics of patient groups based on MAGE in the first 24 hours are shown in Table 2. Patients in the highest MAGE group were older, more likely to be DM, had more higher levels of HbA1c, GA, and cTNI than those in the lower MAGE group In addition, Patients in the highest MAGE group had higher incidences of triple vessels and multivessel disease, lower LVEF, higher MBG.A MAGE profile using the 3 days mean data in diabetic subjects was shown in Figure 1, the 3 days MAGE mean values were 3.90 ± 1.1 mmol/l, 3.34 ± 1.25 mmol/l, 3.16 ± 1.15 mmol/l, respectively (p = 0.001). Figure 2 showed the MAGE profile using the 3 days mean data in nondiabetic subjects, the 3 days MAGE mean values were 2.80 ± 1.6 mmol/l, 2.43 ± 1.62 mmol/l, 2.39 ± 1.49 mmol/l, respectively (p = 0.034).

**Table 2:** Baseline characteristics in AMI patients according to MAGE

**MAGE (mmol/L)**	**<2.37**	**2.37-3.65**	**>3.65**	**P-value**
Subject number	80	78	79	NA
Age (years)	51 ± 13	53 ± 15	57 ± 16	**0.034**
Males	55 (69)	53 (68)	57 (72)	0.831
BMI, kg/m^2^	24.7 ± 3.9	25.1 ± 4.2	25.4 ± 4.5	0.575
LVEF, %	55.8 ± 7.5	52.5 ± 8.1	51.2 ± 8.4	0.001
Systolic BP (mm Hg)	119 ± 21	121 ± 27	123 ± 25	0.533
Diastolic BP (mm Hg)	70 ± 11	69 ± 12	71 ± 15	0.619
Serum creatinine, mg/dL	0.79 ± 0.25	0.80 ± 0.23	0.82 ± 0.27	0.745
Total Cholesterol (mmol/l)	4.61 ± 1.15	4.63 ± 1.18	4.67 ± 1.09	0.387
Triglycerides (mmol/l)	2.20 ± 1.18	2.18 ± 1.35	2.31 ± 1.43	0.802
HbA1c (%)	6.1 ± 1.4	6.4. ± 1.3	7.0 ± 1.5	<0.001
Glycated albumin (%)	11.8 ± 6.5	16.8 ± 7.7	19.9 ± 8.5	<0.001
Peak CK-MB (ng/ml)	35.1 ± 11.6	37.2 ± 13.4	39.5 ± 14.5	0.113
Peak cTNI (ng/ml)	35.3 ± 12.7	36.9 ± 12.5	40.2 ± 11.4	0.038
MBG (mmol/l)	6.9 ± 1.2	8.1 ± 2.1	9.5 ± 1.9	<0.001
**Risk factors (n, %)**				
Hyperlipidemia	28 (35)	31 (40)	34 (43)	0.580
Hypertension	27 (34)	35 (45)	33 (42)	0.337
Current smoking	31 (39)	33 (42)	42 (53)	0.164
Family history	8 (10)	9 (12)	12 (15)	0.592
Obesity	14 (18)	18 (23)	23 (29)	0.222
Diabetes	11 (14)	20 (26)	42 (53)	<0.001
**Previous medication [n (%)]**				
Aspirin	67 (84)	66 (85)	69 (87)	0.801
ACEI or ARB	45 (56)	48 (62)	50 (63)	0.639
β-Blockers	38 (48)	36 (46)	40 (51)	0.847
Statin	37 (46)	39 (50)	41 (52)	0.769
**Angiographic data [n (%)]**				
Single vessel	38 (48)	32 (41)	22 (28)	0.035
Double vessels	27 (34)	26 (33)	24 (30)	0.885
Triple vessels	15 (19)	20 (26)	33 (42)	0.005
Main stem involved	8 (10)	11 (14)	13 (16)	0.483
Multivessel	42 (66)	46 (72)	57 (81)	0.037
TIMI-3 flow after PCI	74 (93)	73 (94)	71 (90)	0.678

Data given as mean ± SD or n (%).

BMI, body mass index; LVEF, left ventricular ejection fraction; LDL-C, Low density lipoprotein-cholesterol; HDL-C, High density lipoprotein-cholesterol; HbA1c, hemoglobin A1c; CK-MB, creatine kinase-MB; cTnI, cardiac troponin I; FBG, fasting blood glucose; OHA, oral hypoglycaemic agent; MBG, Mean blood glucose; MAGE, the mean amplitude of glycemic excursions; SDBG, the standard deviation of blood glucose values.

**Figure 3:**
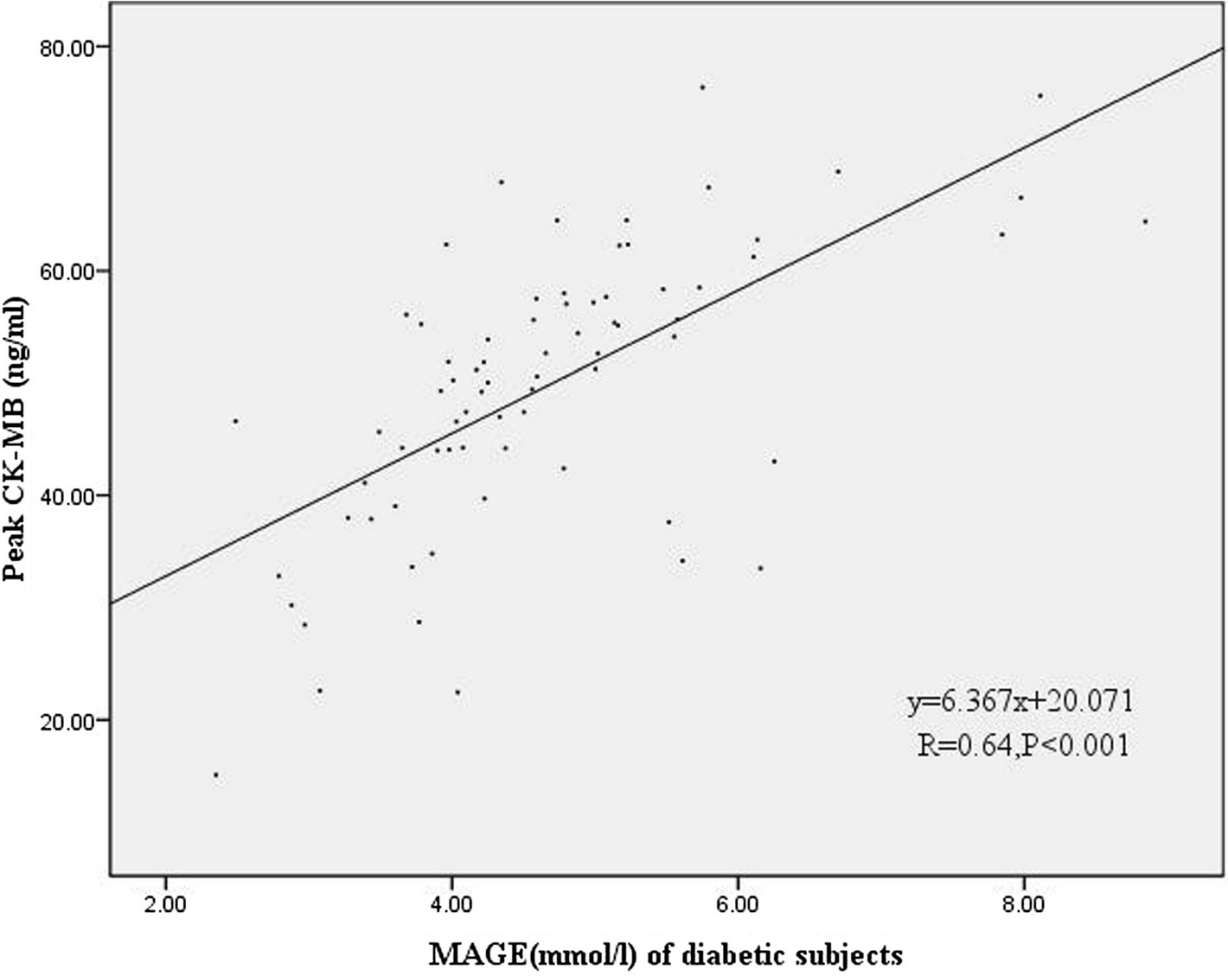
Correlation of peak CK-MB values and MAGE values of the first day in diabetic subjects.

**Figure 4:**
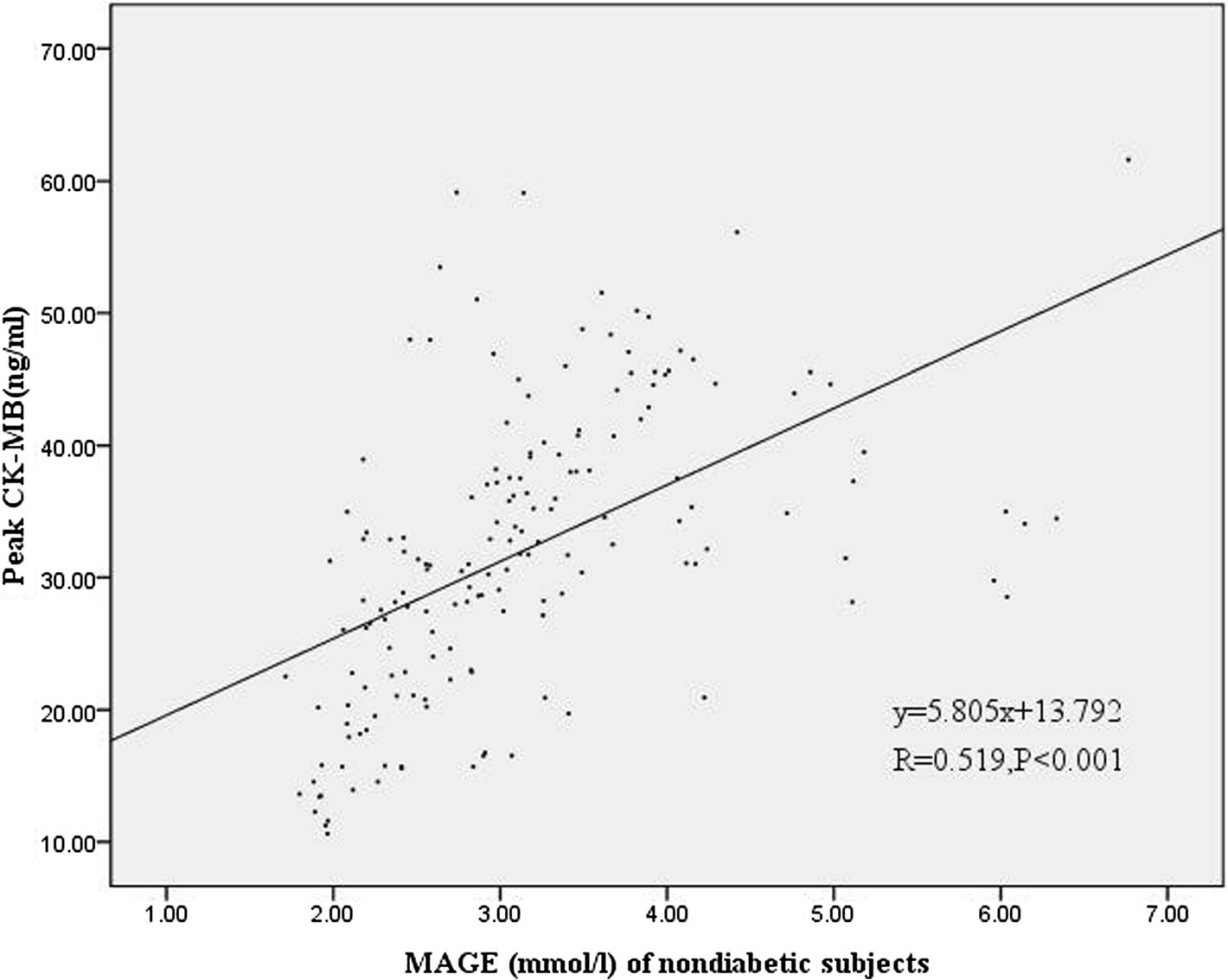
Correlation of peak CK-MB values and MAGE values of the first day in nondiabetic subjects.

**Figure 5:**
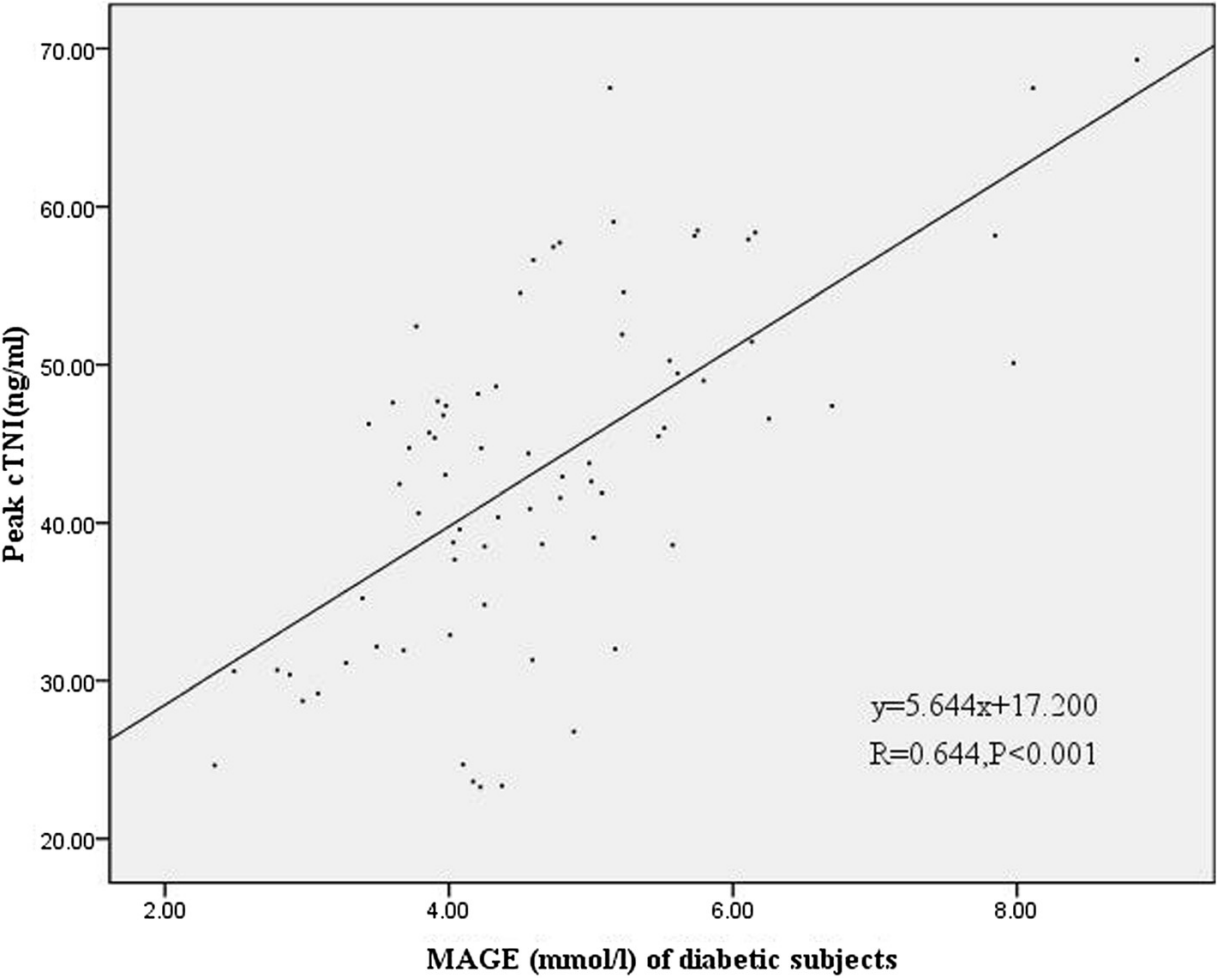
Correlation of peak cTnI values and MAGE values of the first day in diabetic subjects.

**Figure 6:**
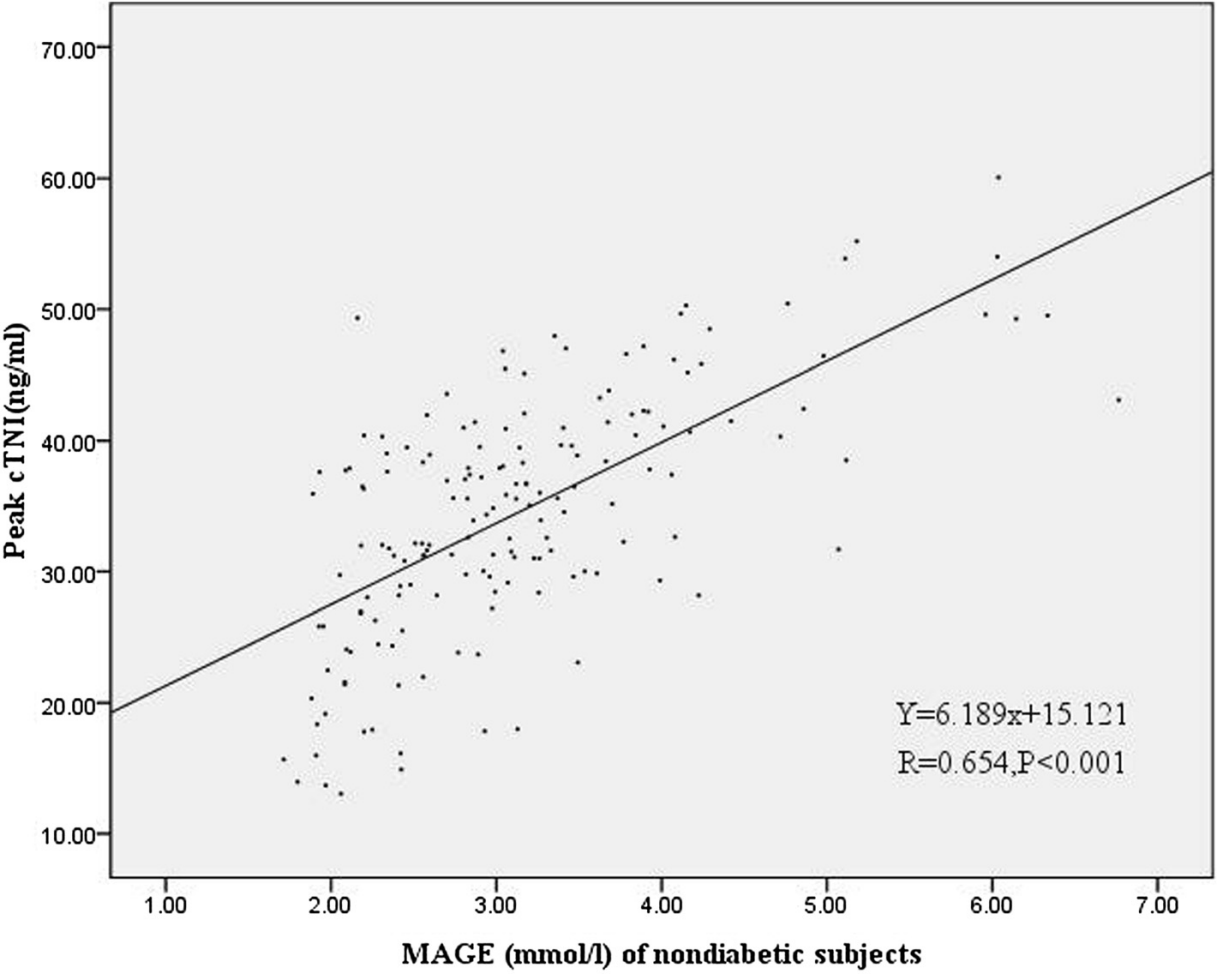
Correlation of peak cTnI values and MAGE values of the first day in nondiabetic subjects.

Figures 3 and 4 showed a statistically significant correlation between peak CK-MB values and MAGE values of the first day in diabetic subjects (r = 0.64, p <0.001) and nondiabetic subjects (r = 0.519, p < 0.001). Moreover, Figures 5 and 6 also revealed a statistically significant correlation between peak cTnI values and MAGE values of the first day in diabetic subjects (r = 0.644, p < 0.001) and nondiabetic subjects (r = 0.654, P <0.001). Compared with non-diabetes patients, DM subjects had higher incidence of composite MACE (24.7% vs. 10.4%, p = 0.004), recurrent angina (12.3% vs. 3.7%, p = 0.025), and non-IRA revascularization (37% vs. 16%, p = 0.001) (Table 3).

**Table 3:** Clinical Outcome during in-hospital and 30-day follow-up in diabetic and non-diabetic patients

		**DM**	**Non-DM**	**P**
Subject number (n)	237	73	164	NA
Composite MACE	35 (14.8)	18 (24.7)	17 (10.4)	0.004
Cardiac death	4 (1.7)	2 (2.7)	2 (1.2)	0.770
Reinfarction	3 (1.3)	2 (2.7)	1 (0.6)	0.469
Repeated TVR	3 (1.3)	1 (1.4)	2 (1.2)	0.594
Recurrent angina	15 (6.3)	9 (12.3)	6 (3.7)	0.025
Malignant arrhythmia	10 (4.2)	5 (6.8)	5 (3)	0.320
Non-cardiac death	1 (0.4)	0 (0)	1 (0.6)	0.678
Non-IRA revascularization	53 (22.4)	27 (37)	26 (16)	0.001

Data given as mean ± SD or n (%).

MAGE, the mean amplitude of glycemic excursions; MACE, major adverse cardiac events; TVR, target vessel revascularization; IRA, infarct related arteries.

**Figure 7:**
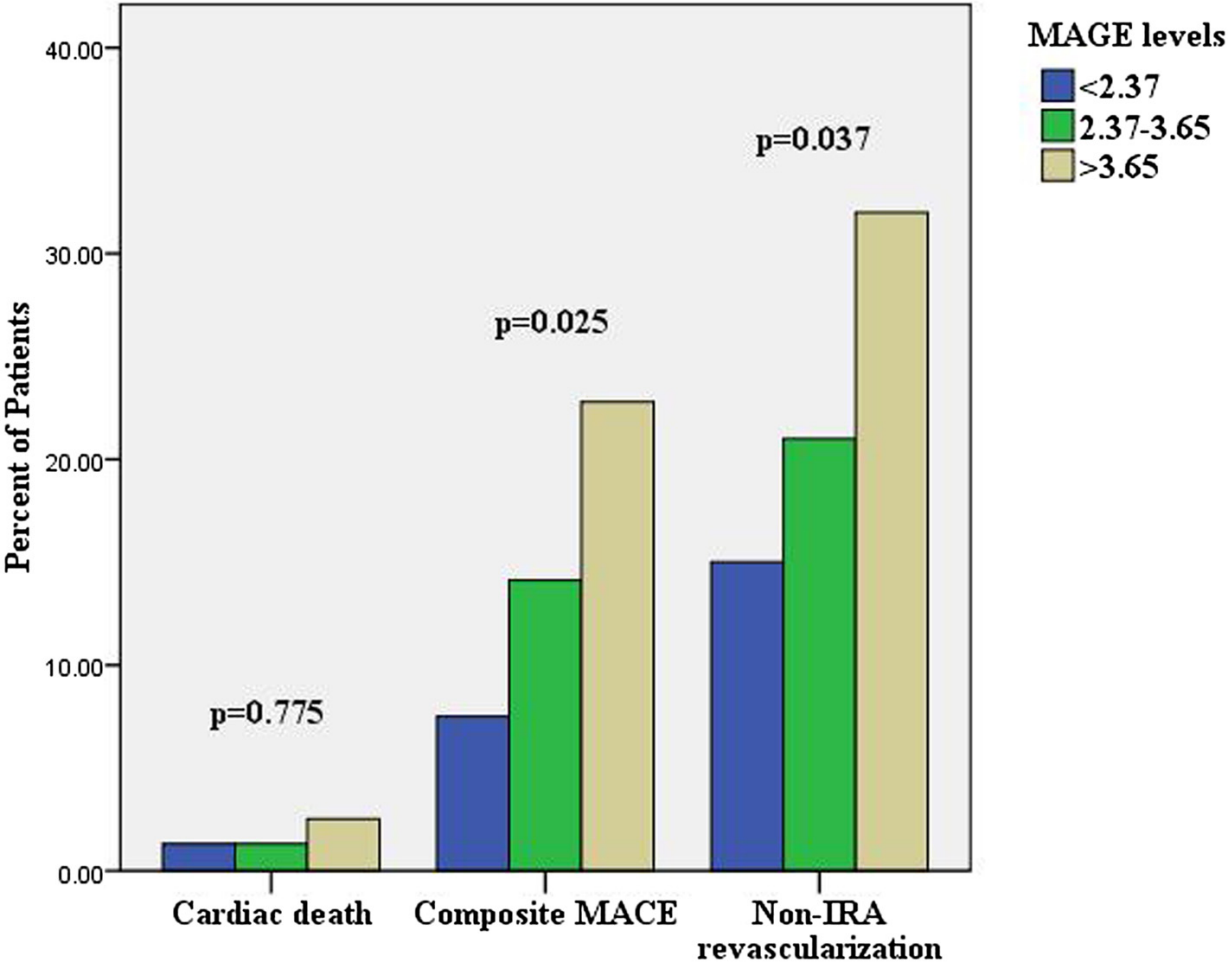
Incidence of MACE during in-hospital and 30-day follow-up.

Table 4 and Figure 7 revealed the clinical outcome of study patients during in-hospital and 30-day follow-up. The results showed 4 patients had died (1.7%) for cardiac causes, 3 patients had reinfarction (1.3%), and 3 patients had repeat TVR (1.3%), 15 patients had recurrent angina(6.3%), 10 patients had malignant arrhythmia (4.2%), 1 patients had died for non-cardiac causes (0.4%), 53 patients had non-IRA revascularization (22.4%). As expected, STEMI patients with MAGE level >3.65 mmol/L had significantly higher incidence of composite MACE compared with STEMI patients with MAGE level from 2.37 mmol/L to 3.65 mmol/L, or < 2.37 mmol/L (22.8% vs. 14.1% vs. 7.5%, p = 0.025). STEMI patients with a higher MAGE level had a significantly higher non-IRA revascularization compared with those with lower MAGE levels (32% vs. 15% vs. 21%, p = 0.037).

**Table 4:** Clinical Outcome of study patients during in-hospital and 30-day follow-up based on MAGE levels

**MAGE (mmol/L)**		**<2.37**	**2.37-3.65**	**>3.65**	**P**
Subject number (n)	237	80	78	79	NA
Composite MACE	35 (14.8)	6 (7.5)	11 (14.1)	18 (22.8)	0.025
Cardiac death	4 (1.7)	1 (1.3)	1 (1.3)	2 (2.5)	0.775
Reinfarction	3 (1.3)	0 (0)	1 (1.3)	2 (2.5)	0.361
Repeated TVR	3 (1.3)	0 (0)	1 (1.3)	2 (2.5)	0.361
Recurrent angina	15 (6.3)	3 (3.8)	5 (6.4)	7 (8.9)	0.416
Malignant arrhythmia	10 (4.2)	2 (2.5)	3 (3.8)	5 (6.3)	0.477
Non-cardiac death	1 (0.4)	0 (0)	1 (1.3)	0 (0)	0.359
Non-IRA revascularization	53 (22.4)	12 (15)	16 (21)	25 (32)	0.037

Data given as mean ± SD or n (%).

MAGE, the mean amplitude of glycemic excursions; MACE, major adverse cardiac events; TVR, target vessel revascularization; IRA, infarct related arteries.

Moreover, compared with DM patients with MAGE level < 2.84 mmol/L, or 2.84 mmol/L to 3.96 mmol/L, DM patients with MAGE level >3.96 mmol/L had significantly higher incidence of composite MACE (8.3% vs. 25% vs. 40%, p = 0.038), and non-IRA revascularization (15% vs. 21% vs. 32%, p = 0.037) (Table 5). However, Table 6 showed non-diabetes patients with a higher MAGE level did not reveal significant differences in the incidence of composite MACE and non-IRA revascularization.

**Table 5:** Clinical Outcome of diabetes subjects based on MAGE levels during in-hospital and 30-day follow-up

**MAGE (mmol/L)**		**<2.84**	**2.84-3.96**	**>3.96**	**P**
Subject number (n)	73	24	24	25	NA
Composite MACE	18 (24.7)	2 (8.3)	6 (25)	10 (40)	0.038
Non-IRA revascularization	27 (37)	4 (15)	10 (21)	13 (32)	0.032

Data given as n (%).

MAGE, the mean amplitude of glycemic excursions; MACE, major adverse cardiac events; TVR, target vessel revascularization; IRA, infarct related arteries.

**Table 6:** Clinical Outcome of non-diabetes subjects based on MAGE levels during in-hospital and 30-day follow-up

**MAGE (mmol/L)**		**<2.21**	**2.21-3.38**	**>3.38**	**P**
Subject number (n)	164	55	55	54	NA
Composite MACE	17 (10.4)	4 (7.3)	6 (10.9)	7 (13)	0.614
Non-IRA revascularization	26 (15.9)	7 (12.7)	9 (16.4)	10 (18.5)	0.704

Data given as mean ± SD or n (%).

MAGE, the mean amplitude of glycemic excursions; MACE, major adverse cardiac events; TVR, target vessel revascularization; IRA, infarct related arteries.

### Multivariate analysis of glucose variability and MACE

Multivariable logistic regression analysis was used to assess whether the association between MAGE and composite MACE was independent of other factors in diabetic and nondiabetic subjects. We chose variables that were significantly different between MAGE categories as co-factors. Include variables were: age, LVEF, HbA1c, GA, Peak cTNI, MBG, and MAGE. The independent predictors of MACE in diabetic subjects were: LVEF (OR 0.829, 95% CI 0.702-0.980, p = 0.028), MBG (OR 2.076, 95% CI 1.077-4.003, p = 0.029), and MAGE (OR 2.857, 95% CI 1.143- 7.139, p = 0.025). Other factors were not significantly associated with MACE: Age (OR 1.055, 95% CI 0.952-1.169, p = 0.310), GA (OR 1.309, 95% CI0.847-2.023, p = 0.225), peak cTNI (OR 1.064, 95% CI 0.726-1.559, p = 0.751), and HbA1c (OR 1.337, 95% CI 0.713-2.507, p = 0.365) (Table 7). The independent predictors of MACE in nondiabetic subjects were: MBG (OR1.627, 95% CI 1.077-2.458, p = 0.021), and MAGE (OR 2.097, 95% CI 1.093-4.026, p = 0.026). Other factors were not significantly associated with MACE (Table 8).

**Table 7:** Multivariable logistic regression to evaluate independent factors on composite MACE in diabetic subjects

**Variable**	**OR**	**95% confidence interval**	**P**
Age	1.055	0.952-1.169	0.310
LVEF	0.829	0.702-0.980	0.028
Peak cTNI	1.064	0.726-1.559	0.751
HbA1C	1.337	0.713-2.507	0.365
GA	1.309	0.847-2.023	0.225
MBG	2.076	1.077-4.003	0.029
MAGE	2.857	1.143-7.139	0.025

OR = Odds ratio; other abbreviations the same as for Tables 1, 2 and 3.

**Table 8:** Multivariable logistic regression to evaluate independent factors on composite MACE in nondiabetic subjects

**Variable**	**OR**	**95% confidence interval**	**P**
Age	1.025	0.940-1.117	0.582
LVEF	0.914	0.803-1.041	0.176
Peak cTNI	1.063	0.810-1.395	0.661
HbA1C	1.244	0.696-2.225	0.461
GA	1.121	0.857-1.467	0.403
MBG	1.627	1.077-2.458	0.021
MAGE	2.097	1.093-4.026	0.026

OR = Odds ratio; other abbreviations the same as for Tables 1, 2 and 3.

## Discussion

In-hospital hyperglycemia portends a poor prognosis in AMI patients [18-24]; however, the prognostic significance of GV after AMI remains controversial. Some studies showed that GV was related to cardiovascular risk [18-20]. However, other studies did not showed similar results. The reanalysis of the Diabetes Control and Complications Trial (DCCT) and DCCT/Epidemiology of Diabetes Interventions and Complications (EDIC) dataset examining the predictive value of GV on microvascular and neurologic complications did not show an effect of GV independent from mean glucose and HbA1c [21-23]. The results of reanalysis of the HEART2D study showed a decrease in GV did not reduce cardiovascular events in patients with type 2 DM after AMI [24]. These previous studies focused on diabetes patients and did not included non-diabetes subjects, moreover, many diabetic patients were treated with insulin and insulin per se could inhibit oxidative stress. It might be possible that these negative results were explained by type 2 diabetic patients with advanced atherosclerosis and patients with diabetes, in contrast with critically ill patients without previously diagnosed diabetes, were not affected by GV because of the ability of cells to adapt to the harmful effects of changing ambient glucose [7] and insulin per se neutralize the deleterious effects of GV on oxidative stress in patients treated with it [25].

One prior study evaluated the role of GV on in-hospital mortality of patients with AMI. The results of this study showed although greater GV was associated with increased risk of in-hospital mortality in patients with AMI in unadjusted analyses, GV was no longer independently predictive after controlling for multiple patient factors [26]. In addition, subgroup analyses by STEMI versus NSTEMI status showed that higher GV was associated with significantly increased mortality risk in patients with STEMI but not in patients with NSTEMI [26]. Su G et al. reported that the early in-hospital GV was an important predictor of mortality and MACE even stronger than HbA1c in elderly patients after AMI [27]. Whether GV plays a special role in patients with STEMI or whether it is simply a prognostic marker remains unclear. So our aim is to assess the prognostic value of GV in STEMI patients undergoing p-PCI. The results of our study showed GV was associated with Composite MACE and non-IRA revascularization. However, our findings, from the subgroup analysis, suggested diabetic subjects with a higher MAGE level had significantly higher incidence of composite MACE and non-IRA revascularization compared with those with lower MAGE levels, but non-diabetic subjects did not show the similar results. These results remained controversial. A systematic review of glycemic variability and complications in patients with diabetes mellitus showed a significant positive association between glucose variability and the development or progression of diabetic retinopathy, cardiovascular events and mortality, and glucose variability could be a predictor of diabetic complications, independent of HbA1c levels in patients with type 2 DM [19]. Another study thought that a deleterious effect resulting from increased glycemic variability was noted among non-diabetic patients, but not among patients with diabetes [28]. In our study the number of study patients was relatively small and the clinical follow-up time was short, which maybe impact the results and need further research in the future. Moreover, multivariable logistic regression analysis showed MAGE, MBG were independent predictors of MACE in diabetic and nondiabetic subjects and HbA1c and GA were not.

CGMS could be the best method to show trends and predict impending glucose excursions and to monitor GV. MAGE was considered as the “gold standard” of glycemic variability [4]. In our study MAGE was calculated through CGMS data. Our study demonstrates that elevated MAGE is an independent predictor of increased risk of MACE in AMI patients. There were major differences in baseline characteristics according to MAGE level. Patients with higher MAGE had more cardiovascular risk factors, such as older age, diabetes. Moreover, patients with higher MAGE had higher peak CK-MB values and peak cTNI values, there was also a clear correlation between MAGE and peak CK-MB values or peak cTNI values, which reflect the severity and size of myocardial necrosis. Angiographic data showed triple vessels and multivessel lesions were more common in patients with higher MAGE. These results indicate that AMI patients with higher GV may be associated with poorer outcomes.

Stress hyperglycaemia is common in acute myocardial infarction, whereas increased catecholamine levels result in decreased insulin secretion and increased insulin resistance [29]. Although stress-induced hyperglycaemia could partly explain the relation between admission GV and outcomes, especially for AMI patients, glycemic excursion itself can also be harmful. Ceriello et al. reported that intermittent hyperglycaemia induced a higher degree of apoptosis in endothelial cells than chronic hyperglycaemia [30]. Quagliaro et al. showed that the apoptosis of endothelial cells exposed to intermittent hyperglycaemia may be related to a reactive oxygen species (ROS) overproduction, through protein kinase C (PKC)-dependent activation of nicotinamide adenine dinucleotide phosphate (NADPH)-oxidase [31]. Both acute and chronic blood glucose variability can induce oxidative stress and chronic inflammation. Moreover, severe glycemic disorders may adversely affect sympathetic dysfunction which is associated with mortality and morbidity of cardiovascular disease [32]. Following the use of CGMS the GV can be accurately and conveniently calculated. Although the prognostic value of GV in AMI patients remains controversial, but GV may be an important predictor of mortality and MACE after AMI. Further studies are needed to investigate the impact of GV on long-term prognosis for STEMI patients and NONSTEMI patients.

### Study limitations

There are several limitations to this study. First, the number of patients was relatively small, so that comparisons of some subgroups might lack power to detect significant differences for selected variables. Second, although we had avoided glucose infusion during CGMS monitoring period, but due to dietary irregular and could not analyze the postprandial BG levels. Moreover, some other factors, such as physical and emotional factors, which maybe affect the result of GV. Third, we are not enrolled all STEMI patients, only enrolled STEMI patients undergoing p-PCI, so maybe miss many critical AMI patients and affect the outcomes. Hence the results of the present study should be interpreted with caution.

## Conclusions

At present the prognostic value of GV in AMI patients remains unclear, our study has revealed that in STEMI patients undergoing p-PCI greater GV is associated with composite MACE and non-IRA revascularization during in-hospital and 30-day follow-up, especially for diabetic subjects. Further studies are needed to investigate whether reducing GV will reduce cardiovascular risk independently from already recognized risk factors and observe the impact of GV on long-term prognosis for STEMI patients undergoing p-PCI.

## Abbreviations

STEMI ST: Segment elevation myocardial infarction; p-PCI: Primary percutaneous coronary interventions; MAGE: Mean amplitude of glycemic excursions; GV: Glycemic variability; CGMS: Continuous glucose monitoring system; MACE: Major adverse cardiac events; SMBG: Self-monitoring of blood glucose; BMI: Body mass index; LVEF: Left ventricular ejection fraction; LDL-C: Low density lipoprotein-cholesterol; HDL-C: High density lipoprotein-cholesterol; CK-MB: Creatine kinase-MB; cTnI: Cardiac troponin I; FBG: Fasting blood glucose; MBG: Mean blood glucose; SDBG: The standard deviation of blood glucose values.

## Competing interests

The authors declare that they have no competing interests.

## Authors’ contribution

Conceived and designed the experiments: Y-jZ and S-wY. Performed the experiments: J-wZ. Analyzed the data: J-wZ and L-jH. Contributed reagents/materials/analysis tools: S-jC and QY. Wrote the paper: J-wZ. All authors have contributed significantly, and all authors read and approved the final manuscript.
